# The meta-analysis^☆^

**DOI:** 10.1016/j.bjorl.2017.06.001

**Published:** 2017-06-09

**Authors:** Fernando de Andrade Quintanilha Ribeiro

**Affiliations:** Faculdade de Medicina da Santa Casa de São Paulo, São Paulo, SP, Brazil

In 1543, a meta-analysis was proposed by the Catholic Church to confirm or deny the theory of geocentrism (i.e., the Earth as the center of the universe) as mentioned in the scriptures. There were rumors that one individual named Nicolaus Copernicus, a Pole, was suggesting that the sun, and not the earth, was the center of the universe.

The meta-analysis was commissioned by Cardinal St. Robert Belarmino (Roberto Francesco Romolo Bellarmino), of the Holy Inquisition, to famous statisticians, at the request of Pope Clement VII, aiming to elucidate the matter once and for all.

The main works were those by Greek philosopher and scientist Aristotle, written around 300 BC, and those of Greek astronomer, geographer, and mathematician Hipparchus in 190 BC, as well as many others that preceded it, such as those by Sumerian, Babylonian, Egyptian, Chinese, and Indus Valley astronomers (Mayan astronomy was not yet known). The works that came after them, such as those by Claudius Ptolemy, in Alexandria in 90 BC and those by the Arab astrologer Albumasar in 886 AD, among others less known but no less important, were also collected. The works had been carried out over a period of 2500 years.

For an adequate meta-analysis, all the works dealt with the same subject and had the same methodology; that is, the detailed observation of the sky, either by day or by night. Only 285 of the first 1636 works were selected, as many of them were lost between distrustful divine considerations and inadequate translations. It was observed, after statistical and evidence-based calculations, the obvious rotation of the sun, moon, and stars was around the earth. Hence, the numbers did not lie and the church's geocentric theory was proven correct through an unsuspected meta-analysis of hundreds of works. The State of the art in astronomy and geophysics.

At this time, in the midst of the Renaissance, the aforementioned Nicolaus Copernicus was almost arrested for blasphemy. Persecuted and sick, he presented his work “De Revolutionibus Orbium Coelestium”, which does not require translation, and thereafter he soon died.

After his death, all who believed in heliocentrism were fiercely persecuted by the Church. One of them was Giordano Bruno, who was burned alive by the inquisition for not recanting his ideas. Another was Galileo Galilei who, to avoid having the same end, had to swear to Pope Urban VIII that the Earth was the center of the universe and did not move around the sun; he uttered quietly before going to the dungeon: “eppur si move” (translated from the archaic Roman language – but it does move!).

Nonetheless, the world continued to spin and, some time later, Kepler came to confirm and improve upon the concept of heliocentrism.

Well… after that came Isaac Newton, Einstein, NASA, the Big Bang, the Hubble, quantum physics, and who knows what else will come.

## Conclusion

Do not “religiously” believe in all meta-analyses, and do not unconsciously burn the non-believing and divergent ones at the stake.

## Conflicts of interest

The author declares no conflicts of interest.

